# Acute Antibody-Mediated Rejection in Liver Transplant Recipients with Autoimmune Liver Disease: A Clinical and Pathologic Study of 4 Cases

**DOI:** 10.3390/jpm13010041

**Published:** 2022-12-25

**Authors:** Hongmei Jiang, Hui Guo, Bo Yang, Yuanyuan Zhao, Lai Wei, Zhishui Chen, Dong Chen

**Affiliations:** Institute of Organ Transplantation, Tongji Hospital, Tongji Medical College, Huazhong University of Science and Technology; Key Laboratory of Organ Transplantation, Ministry of Education; NHC Key Laboratory of Organ Transplantation; Key Laboratory of Organ Transplantation, Chinese Academy of Medical Sciences, Wuhan 430030, China

**Keywords:** acute antibody-mediated rejection, liver transplantation, autoimmune liver disease

## Abstract

**Background**: Acute antibody-mediated rejection (AMR) is an uncommon complication after ABO-compatible liver transplantation (LT). This case series investigated the clinicopathologic characteristics and outcomes of acute AMR in LT recipients with autoimmune liver disease (ALD). **Patients and Methods**: Among 809 patients who underwent LT from January 2014 to December 2020, four ALD patients developed AMR, which was confirmed based on clinical features, histopathology of liver biopsy, donor-specific antibodies (DSA) or panel reactive antibody (PRA) level. Therapies were individualized based on clinical manifestations. **Results**: The incidence of acute AMR was 0.49%, and the incidence of acute AMR with ALD and non-ALD recipients was 11.1% and 0%, respectively. Three patients had strongly positive HLA class II DSA, and one patient was with the PRA class I and II sensitivities, which were >80%; complement component 4d (C4d) staining was negative in all patients. The first patient underwent re-LT, and the other three patients had good prognoses with treatments. **Conclusions**: ALD patients are prone to acute AMR after LT, thus should be kept vigilant against the occurrence of acute AMR.

## 1. Introduction 

Autoimmune liver disease (ALD) is a rare disease with a reported incidence of less than 50 per 100,000 [1]. Primary biliary cholangitis, primary sclerosing cholangitis, and autoimmune hepatitis are the major types of ALDs [2]. Several drugs can improve the long-term prognosis in patients with early ALD, while for patients with end-stage ALD, liver transplantation (LT) is still the only effective treatment option [3].

The incidence of acute T cell-mediated rejection (TCMR) after LT is between 10% and 30% [4,5]. The incidence of chronic TCMR is about 2~5% in adults but may also reach 8~16% in the pediatric population [6], while the incidence of acute antibody-mediated rejection (AMR) is rare (<5%) [7]. The liver is the only organ resistant to antibody-mediated rejection, even hyperacute rejection [8]. Although the liver belongs to the immunologically privileged organ and acute AMR is rarely observed after LT, it has been recognized as a cause of graft dysfunction in a proportion of LT recipients [9]. Additionally, acute AMR has been widely reported after ABO-incompatible LT, but it is relatively rare after ABO-compatible LT, with a prevalence of 0.8% to 3.6% [10]. The diagnosis of acute AMR is challenging, and so far, no guidelines and consensus criteria have been proposed [11,12].

Studies have shown that LT recipients with ALD have a higher incidence of acute TCMR than other LT recipients [13]. Previous studies have discussed a correlation between ALD and TCMR after LT [13,14]. Herein, we discussed clinical features in four LT recipients with ALD that developed acute AMR.

## 2. Patients and Methods

A total of 809 patients underwent primary liver transplantation from January 2014 to December 2020 performed by our medical team, including 36 patients with ALD (4 cases had postoperative acute AMR) and 773 patients without ALD and postoperative acute AMR (these data are only for the adult recipients). Four patients with end-stage liver disease caused by ALD (Patient 1 and Patient 3 had autoimmune hepatitis; Patient 2 and Patient 4 had primary biliary cholangitis) developed acute AMR within 30 days after ABO-compatible LT. Acute AMR was diagnosed based on the following four criteria: (i) clinical signs of graft dysfunction; (ii) histopathology indicative of acute injury ± positive C4d stain; (iii) presence of PRA or human leukocyte antigen (HLA) DSA; (iv) reasonable exclusion of other lesions that might cause a similar pattern of injury. Biochemical indicators of liver function (including alanine aminotransferase (ALT), aspartate aminotransferase (AST), total bilirubin (TBIL), and direct bilirubin (DBIL)), hepatic histopathology, PRA, HLA DSA, immunosuppressive regimen, and prognosis were evaluated for each patient.

## 3. Histopathology

Percutaneous liver biopsy guided by ultrasound was performed when graft dysfunction was clinically indicated. All liver allograft biopsies were analyzed by an experienced liver pathologist and reviewed by a second pathologist for the purpose of this study, according to Banff Schema on liver allograft rejection. In addition, all liver biopsies were completed with C4d staining in our center. The C4d positive staining criteria were vascular endothelial cells (>50% lumen) positive staining, including hepatic sinusoidal endothelial cells and portal interstitial microvessels, interlobular vein, and central vein endothelial cells staining [15,16].

## 4. HLA Antibodies Detection

HLA antibody detection was performed using Luminex-bead-based panel reactive screening immunoassays. Detection of HLA antibodies in patients, donor-recipient mismatched HLA comparisons, and DSA positivity with potential clinical significance is preliminarily defined as mean fluorescence intensity (MFI) ≥5000; yet, the positive cutoff values vary from laboratory to laboratory and need to be standardized [17].

## 5. Immunosuppression Management

Four LT recipients received an immunosuppressive regimen with steroids, mycophenolate mofetil (MMF), and tacrolimus in the early post-LT period. Basiliximab (20 mg twice, 2 h before surgery and 4 days after surgery) was used for immune induction. The valley of tacrolimus troughs levels varies based on each patient’s clinical condition, with an initial target valley level of 6–7 ng/mL (during the first two weeks of treatment); the actual tacrolimus level was 4–6 ng/mL at the time that AMR was diagnosed. Acute AMR therapies were individualized, and included steroids, ATG (25 mg, Qd), high-dose IVIG (20 g, Qd), rituximab or bortezomib (1.3 mg/m^2^), and plasmapheresis (every other day, the duration for 1.5–2 h).

### 5.1. Patient 1 

**Figure 1:**
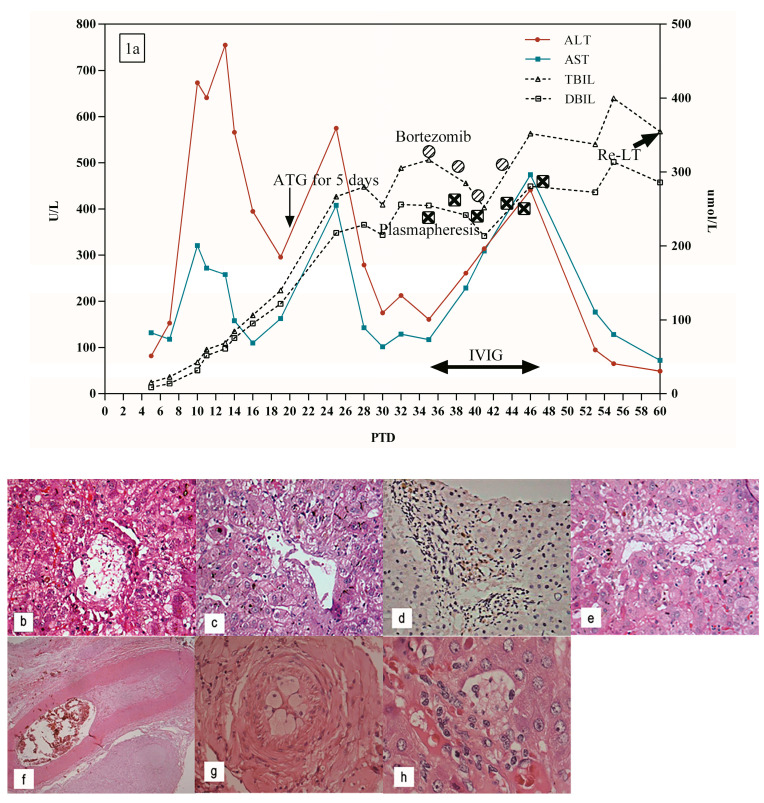
(**a**) Clinical course of patient 1. (**b**) Portal inflammatory infiltrate, slight bile ductular reaction, and hepatocyte swelling and necrosis around the central vein on PTD 20 (hematoxylin-eosin (H&E) × 400). (**c**) Aggravation of bile duct cholestasis, severe hepatocyte necrosis around the central vein on PTD 35 (H&E × 400). (**d**) Immunocytochemistry shows negative C4d staining in the portal vein and capillaries on PTD 35 (×400). (**e**) Further aggravation of central perivenulitis, bile duct cholestasis, and hepatocyte necrosis on PTD 60 (H&E×400). (**f**) H&E × 100, (**g**) H&E × 400, (**h**) H&E × 1000) Intrahepatic arterial endothelial edema, lumen stenosis, even occlusion, central perivenulitis, severe hepatocyte necrosis around the central vein in resected transplanted liver on PTD 62.

A 48-year-old woman with end-stage liver disease caused by ALD underwent piggyback LT in April 2014. One week after the transplant, the ALT (153 U/L) and AST (118 U/L) began to increase, while bilirubin levels were normal (Figure 1a). The Doppler ultrasound of the liver was normal, and the biopsy revealed few lymphocytes infiltrates in the portal area, slight hepatocyte swelling, and necrosis, and slight cholestasis of capillary bile ducts around the central vein compatible with Banff Schema (Figure 1b), so mild ischemia-reperfusion injury or drug-induced liver injury was considered. To avoid hepatotoxicity of the drugs of tacrolimus, the patient was treated with ATG therapy for 5 days (Figure 1a), during which tacrolimus was discontinued. After treatment, the ALT, AST, and bilirubin levels remained elevated.

The second liver biopsy showed the lymphocyte infiltrates in the portal area, cholestasis of the capillary bile ducts, and hepatocyte necrosis around the central vein was more severe than before (Figure 1c). C4d stain was negative (Figure 1d). No significant biliary stricture or dilation was observed by magnetic resonance cholangiopancreatography (MRCP). HLA antibody testing showed strongly positive DSA to DQ7 (8307.19 MFI). So, the patient was diagnosed with acute AMR and treated with high doses of IVIG, bortezomib, and plasmapheresis (Figure 1a). However, the ALT and AST fluctuated back and forth during this period (they did not reach normality), while bilirubin levels continued to rise. 

The third liver biopsy showed that the inflammatory infiltration in the portal area was relieved, but focal necrosis of hepatocytes around the central vein, further aggravation of central perivenulitis and bile duct cholestasis could be seen (Figure 1e). The liver biochemistry was still abnormal, and cholestasis continued to worsen. In order to save her life, she received a re-LT. The pathological findings of the resected transplanted liver showed obvious intrahepatic arterial endothelial edema, lumen stenosis, even occlusion, central perivenulitis, and severe hepatocyte necrosis around the central vein, which further confirmed the occurrence of AMR (Figure 1f–h). The patient was followed for 1 year without any adverse events.

### 5.2. Patient 2

**Figure 2:**
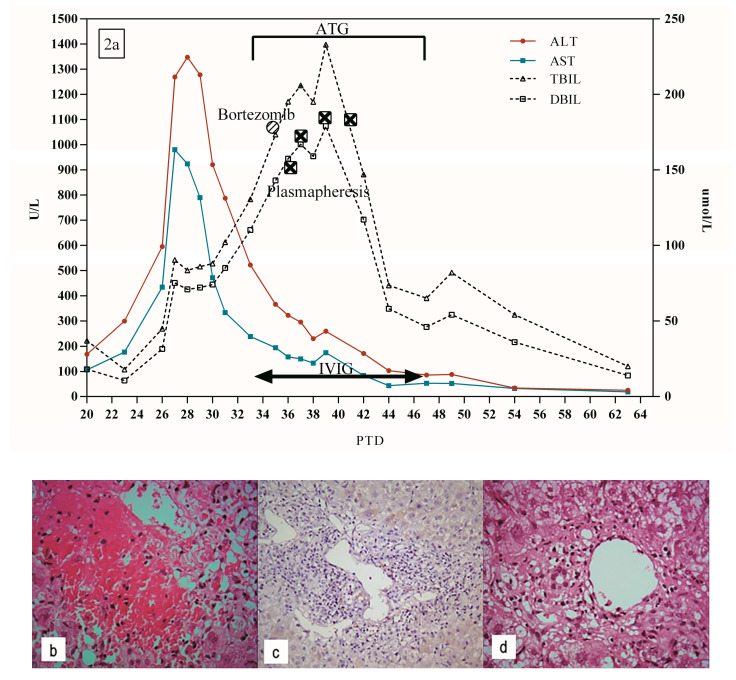
(**a**) Clinical course of patient 2. (**b**) Moderate to severe central perivenulitis, hepatocyte hemorrhage, and necrosis around central vein on PTD 30 (H&E × 400). (**c**) Negative C4d staining on PTD 30 (×200). (**d**) Mild central perivenulitis on PTD 51 (H&E × 400).

A 34-year-old male with end-stage liver disease caused by ALD received ABO-identical orthotopic LT on May 2017. The AST and ALT began to rise on PTD 20 (Figure 2a). The Doppler ultrasound and MRCP of the liver graft were both normal. However, due to persistent elevation in AST 924 U/L and ALT 1348 U/L, a liver biopsy was performed. Histopathology showed lymphocyte infiltrates in the portal area, moderate to severe central perivenulitis, hepatocyte hemorrhage, and necrosis around the central vein (Figure 2b). C4d staining was negative (Figure 2c). Acute AMR was suspected, so he was treated with steroids, IVIG, and ATG. Yet, liver function did not improve after treatment (Figure 2a). Strongly positive DSA to DQ2 (9907.12 MFI) combined histopathology confirmed AMR in the liver graft. The patients continued treatment for acute AMR with plasmapheresis, bortezomib, IVIG, and ATG (Figure 2a). The biochemical indexes of liver function began to gradually decrease. After treatment, liver biopsy showed inflammatory portal infiltration and central perivenulitis were significantly relieved, and hepatocyte hemorrhage and necrosis around the central vein were significantly reduced (Figure 2d). Terminally, the levels of ALT, AST, and bilirubin decreased to normal 63 days after LT.

### 5.3. Patient 3

**Figure 3:**
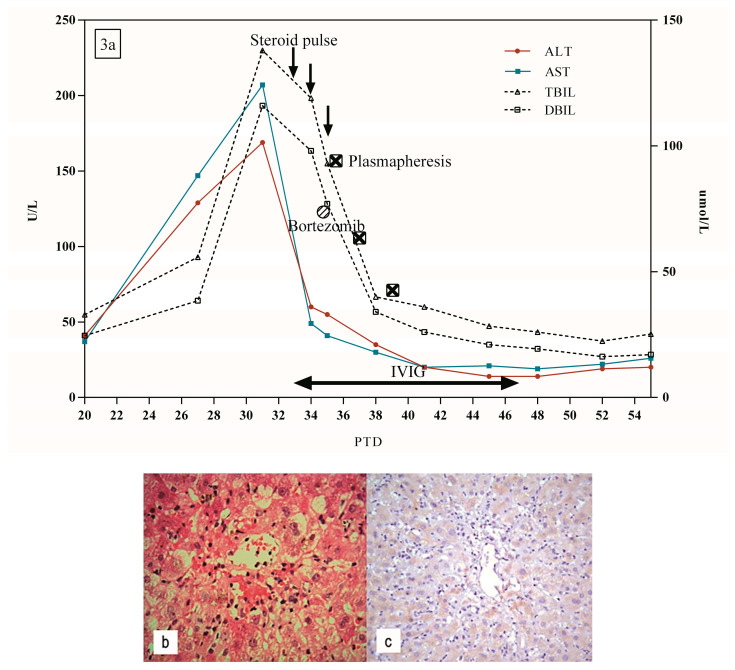
(**a**) Clinical course of patient 3. (**b**) Mild central perivenulitis and inflammatory portal infiltration on PTD 33 (H&E × 400). (**c**) Negative C4d staining on PTD 33 (×200).

A 48-year-old female with end-stage liver disease caused by ALD underwent ABO-compatible piggyback LT in December 2017. The patient was admitted on PTD 27 due to an elevated AST (147 U/L) and ALT (129 U/L) (Figure 3a). Doppler ultrasound and MRCP were normal. After conservative treatment, she had continued elevation of AST, ALT, TBIL, and DBIL, which peaked on PTD 31 at 207 U/L, 169 U/L, 138 umol/L, and 116 umol/L, respectively. She was treated with steroids (80 mg/day) for two days, yet no significant decrease in the biochemical indexes of liver function was observed. 

A liver biopsy revealed mild central perivenulitis and inflammatory portal infiltration (Figure 3b). C4d stain was negative (Figure 3c). PRA of class I and II test were 98.6% and 84.1%, respectively, suggesting acute AMR. The patient then received IVIG, bortezomib, and plasmapheresis treatment (Figure 3a). At the same time, she received steroid therapy (500 mg of methylprednisolone for 3 days; 80 mg prednisone on day 4, and progressively tapered to 10 mg and maintained this dosage afterward). Ultimately, the liver biochemical parameters returned to normal 45 days after LT.

### 5.4. Patient 4

**Figure 4:**
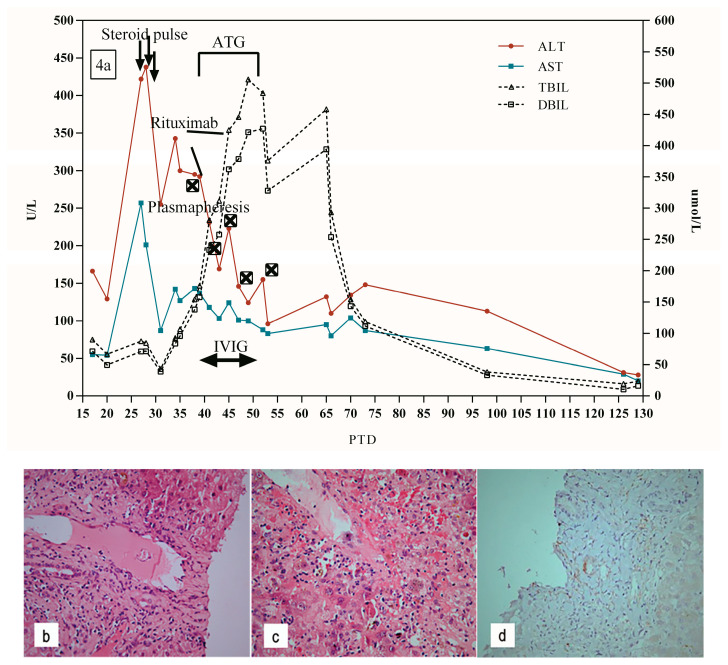
(**a**) Clinical course of patient 4. (**b**) Mild lymphocytic infiltrate in the portal tract, ductular reaction, and scattered hepatocyte necrosis on PTD 28 (H&E × 400). (**c**) Central perivenulitis and hepatocyte necrosis around the central vein furtherly aggravated and lymphocytic infiltrate in the portal tract on PTD 39 (H&E × 400). (**d**) Negative C4d staining on PTD 39 (×200).

A 51-year-old woman with end-stage liver disease caused by ALD underwent orthotopic LT in December 2019. The patient was admitted on PTD 27 due to an elevated AST (257 U/L) and ALT (422 U/L) (Figure 4a). Doppler ultrasound and MRCP were normal. A liver biopsy showed mild lymphocytic infiltrate in the portal tract and scattered hepatocyte necrosis (Figure 4b), so the TCMR was considered. The patient was treated with a steroid pulse (500 mg of methylprednisolone for 3 days). After treatment, the ALT and AST began to decrease, but bilirubin continued to rise. 

On PTD 39, the TBIL and DBIL were 176.2 µmol/L and 157.7 µmol/L, respectively. Besides lymphocytic infiltration in the portal tract, the second liver biopsy showed further aggravated central perivenulitis and hepatocyte necrosis around the central vein (Figure 4c). C4d stain was negative (Figure 4d). In addition, HLA antibody testing showed strongly positive DSA to DQ7 (11126.41 MFI) and DQ9 (10642.11 MFI), which suggested acute AMR.

In the next half month, the patient was given plasmapheresis, combined with rituximab (100 mg, twice with an interval of 5 days), ATG, and IVIG treatment (Figure 4a). During this period, she had continued elevation of her TBIL and DBIL, which peaked at 505.2 µmol/L and 421.2 µmol/L on PTD 49, respectively. Repeat antibody testing showed DSA to DQ7 (7178.65 MFI) and DQ9 (6573.781 MFI) were decreased on PTD 54. On PTD 65, the bilirubin levels began to decrease. Eventually, the ALT, AST, TBIL, and DBIL returned to normal on PTD 126.

## 6. Results

From January 2014 to December 2020, in our medical group, the incidence of acute AMR was 0.49% (4/809), and the incidence rates of acute AMR after LT in ALD and non-ALD recipients were 11.1% (4/36) and 0% (0/773), respectively. The incidence of AMR was significantly higher in patients with ALD than in those without ALD.

Four LT recipients were diagnosed with acute AMR based on the clinical manifestations, histopathological and immunological criteria described above. Simultaneously, the recurrence of ALD was ruled out in all the patients. A summary of their clinical characteristics, histopathology, and outcomes is shown in Table 1.

While treatment modality varied for each patient, all patients were treated with plasmapheresis, which was well tolerated. Because of the delay in diagnosis, patient 1 had acute refractory AMR and received re-LT. The other three patients showed improvement in graft function after therapy. All patients were followed up for 1 year after discharge, and the liver functions were normal. 

## 7. Discussion

The incidence of AMR in ABO-compatible LT is relatively low, and AMR was found in the case reports and reviews to lead to liver allograft dysfunction, and the failure of transplantation is relatively rare [18]. Herein, we reported four patients with ALD who developed acute AMR after LT. Based on our findings, liver dysfunction and hyperbilirubinemia early after surgery can indicate acute AMR, yet the following conditions should be excluded first: ischemia/reperfusion injury, biliary obstruction and/or stenosis, hepatic artery thrombosis, acute TCMR, viral infections, recurrent primary disease [19,20,21]. In this study, patient 1 was diagnosed late with AMR due to an insufficient understanding of the characteristics of AMR and thus missed the optimal treatment time; anti-AMR treatment failed to save the liver graft failure, and re-LT was the only treatment for AMR. The failure of patient 1 suggests that histopathological manifestations of central perivenulitis, hepatocyte hemorrhage, and necrosis around the central vein could be suggestive of acute AMR. Interestingly, these manifestations were found in the other three patients. Yet, in those cases, acute AMR was diagnosed and timely treated, so the other three patients had good prognosis.

According to the 2016 Banff Working Group on Liver Allograft Pathology, the “signature” acute AMR microvascular pathology lesions include endothelial cell enlargement/hypertrophy with dilatation and edema, and endothelitis with intraepithelial and marginating eosinophils, macrophages, lymphocytes, and neutrophils within the portal veins, capillaries, and inlet venules [15]. In addition, acute TCMR histopathology often shows venous endothelial inflammation in the portal tract, bile duct inflammation damage, and portal inflammation. Although the pathological manifestations of acute AMR were more serious in our patients and often involved the centrilobular vein, the most common features included central perivenulitis, hepatocyte hemorrhage, and necrosis around the central vein (Table 1). Therefore, we believe that lymphocyte infiltrates in the portal area, central perivenulitis, hepatocyte hemorrhage, and necrosis around the central vein are outstanding pathological features of acute AMR in liver graft. When the above-mentioned pathological manifestations occur, further C4d staining and DSA examination should be conducted to confirm the occurrence of AMR. 

C4d staining is a key component in diagnosing acute AMR [22]. Meanwhile, diffuse microvascular C4d deposition is part of the Banff standard. However, studies have shown that the positive rate of C4d in LT AMR is only 4.5%, which is far lower than the positive rate of C4d in transplanted kidney biopsy tissue of 15% [23]. Other studies have shown that C4d is more common in chronic inflammatory liver diseases than AMR (54% vs. 3.7%, respectively) [24]. Complements may have different roles in liver rejection because many of the complement components are produced in the liver. Therefore, the liver endothelium has a stronger resistance to complement-induced damage [25]. All the LT recipients in our study were negative for C4d staining, but combined with pathological findings and HLA-DSA, excluding possible causes of a similar injury, all were diagnosed as acute AMR. Furthermore, anti-AMR therapy was highly effective. Therefore, C4d-negative LT recipients can be diagnosed when they meet other diagnostic criteria for acute AMR.

Class II DSA is more associated with graft dysfunction of all organ types. AMR can occur in prefabricated or de novo class I and/or class II DSA [26,27,28]. In our case series, three of four patients with acute AMR were associated with class II DSA (Table 1). Although patient 3 HLA-DSA results were negative, PRA class I and II sensitivities were >80%; thus, it is suggested that non-HLA-DSA antibodies have an important role in AMR. The histopathological damage of patient 3 was mild, and the effect of anti-AMR therapy was remarkable. AMR was confirmed based on the clinical manifestations and evaluation of liver function, liver biopsy results, and effective anti-AMR therapy.

The mainstay of treatment is plasmapheresis and IVIG that theoretically remove and neutralize the antibodies mediating the rejection process [29]. The other modalities experimented with all work against B cells and plasma cells, which are the main cell type in the pathophysiology of AMR. Examples include rituximab (an anti-CD20 antibody, the depletion of naive and memory B cells), bortezomib (inactivation of plasma cells), and eculizumab (the blockade of complement component C5 by monoclonal antibodies) [29]. Yet, bortezomib has been associated with some adverse reactions, such as viral infection, thrombocytopenia, or peripheral neuropathy. Additionally, there is a potential risk of persistent B cell failure and hypogammaglobulinemia when bortezomib is combined with rituximab [30]. Steroids have also been found to reduce the likelihood of de novo DSA, and a combination of tacrolimus and steroids may be effective [31,32]. Because activating B cells requires the assistance of T cells, acute TCMR is also accompanied by the process of AMR, so we think it is necessary to apply ATG or steroids in anti-AMR therapy. In our case series, a combination of steroids, ATG, plasmapheresis, IVIG, and bortezomib anti-AMR therapies appeared to be most efficacious (Table 1). For LT recipients whose anti-AMR treatment cannot save AMR-induced graft injury, the most important thing is to select the donor for re-LT [33]. We suggest evaluating DSA before re-LT to guide clinical management when allograft dysfunction, rejection, or other complications occur after re-LT [12]. The experience of the failure of anti-AMR therapy in patient 1 suggests that acute AMR should be examined and early and timely treated. If the function of the graft cannot be reversed after anti-AMR therapy, re-LT should be considered as soon as possible. 

Our data suggest that ALD patients are prone to acute AMR after LT. In order to prevent the occurrence of acute AMR, ATG or basiliximab immune induction is recommended; ATG or basiliximab induction strategies are often used to delay the introduction of calcineurin inhibitor (CNI) therapy in patients with renal dysfunction. Induction therapy is also used for those at higher immunological risk (re-transplantation for rejection, immune-mediated liver disease, simultaneous liver-kidney; highly sensitized)^7^. Simultaneously, a too-low tacrolimus blood concentration should be avoided and maintained at about 7–10 ng/mL. In order to avoid insufficient immunosuppression, post-transplant maintenance immunosuppression regimens should be tacrolimus combined with MMF and steroids.

The present study has several limitations. This is a retrospective study, and the number of cases with AMR was small. All four recipients with AMR had ALD, and two had autoimmune hepatitis, while the specific type of the other two cases was primary biliary cholangitis.

## 8. Conclusions

Herein, we reported four cases of AMR in ALD patients who underwent LT. AMR was not observed in LT patients without ALD. The main characteristic manifestations of AMR were central perivenulitis, hepatocyte hemorrhage, and necrosis around the central vein in liver biopsy, even if the C4d staining was negative. Combined with DSA or PRA, acute AMR should be timely examined. To sum up, our data suggest that ALD patients are prone to acute AMR after LT.

## Figures and Tables

**Table 1:** Summary of cases of acute AMR following LT.

Patient	Age, Sex	Start of Graft Dysfunction	PRA Class I	PRA Class II	DSA Specificity	Treatment ^a^							Notable Findings on Liver Histopathology	C4d Stain
						Steroids	Plasmapheresis	IVIG	Rituximab	ATG	Bortezomib	AMR Outcome		
1	48 y, F	PTD 7	3.40%	95.90%	DQ 7		✓	✓		✓	✓	Re-LT, survival	PTD 20: portal inflammatory infiltrate, slight bile ductular reaction and hepatocyte swelling and necrosis around the central vein	-
													PTD 35: the aggravation of bile duct cholestasis, severe hepatocyte necrosis around the central vein	
													PTD 60: further aggravation of central perivenulitis, bile duct cholestasis, and hepatocyte necrosis	
													PTD62: intrahepatic arterial endothelial edema, lumen stenosis, even occlusion, central perivenulitis, severe hepatocyte necrosis around the central vein in resected transplanted liver	
2	34 y, M	PTD 20	2.17%	0.31%	DQ 2	✓	✓	✓		✓	✓	Survival	PTD 30: lymphocyte infiltrates in the portal area, moderate to severe central perivenulitis, local hepatocyte hemorrhage and necrosis around central vein	-
													PTD 51: mild central perivenulitis	
3	44 y, F	PTD 27	98.56%	84.05%	NT	✓	✓	✓			✓	Survival	PTD 33: mild central perivenulitis, portal inflammatory infiltration	-
4	51 y, F	PTD 27	42.30%	41.50%	DQ 7, DQ9	✓	✓	✓	✓	✓	✓	Survival	PTD 28: mild lymphocytic infiltrate in portal tract, ductular reaction, and scattered hepatocyte necrosis	-
													PTD 39: central perivenulitis and hepatocyte necrosis around the central vein furtherly aggravated, and lymphocytic infiltrate in portal tract	

F, female; M, male; NT, not tested; y, years old. ^a^ Treatment in addition to standard induction and maintenance immunosuppression.

## Data Availability

The original contributions presented in this study are included in the article. Further inquiries can be directed to the corresponding author.

## Abbreviations

ALD, autoimmune liver disease; AMR, antibody-mediated rejection; ATG, anti-human thymocyte immunoglobulin; ALT, alanine aminotransferase; AST, aspartate aminotransferase; C4d, complement component 4d; CNI, calcineurin inhibitor; DSA, donor-specific antibodies; HLA: human leukocyte antigen; H&E, hematoxylin-eosin; IVIG, intravenous immunoglobulin; LT, liver transplantation; MFI, mean fluorescence intensity; MMF, mycophenolate mofetil; MRCP, magnetic resonance cholangiopancreatography; PRA, panel reactive antibody; TCMR, T cell–mediated rejection; TBIL, total bilirubin; DBIL, direct bilirubin.

## References

[1] Edmunds C., Ekong U.D. (2016). Autoimmune Liver Disease Post-Liver Transplantation: A Summary and Proposed Areas for Future Research. Transplantation.

[2] Floreani A., De Martin S., Secchi M.F., Cazzagon N. (2019). Extrahepatic autoimmunity in autoimmune liver disease. Eur. J. Intern. Med..

[3] Tanaka A., Kono H., Leung P.S.C., Gershwin M.E. (2020). Recurrence of disease following organ transplantation in autoimmune liver disease and systemic lupus erythematosus. Cell. Immunol..

[4] Levitsky J., Goldberg D., Smith A.R., Mansfield S.A., Gillespie B.W., Merion R.M., Lok A.S., Levy G., Kulik L., Abecassis M. (2017). Acute Rejection Increases Risk of Graft Failure and Death in Recent Liver Transplant Recipients. Clin. Gastroenterol. Hepatol..

[5] Wiesner R.H., Demetris A.J., Belle S.H., Seaberg E.C., Lake J.R., Zetterman R.K., Everhart J., Detre K.M. (1998). Acute hepatic allograft rejection: Incidence, risk factors, and impact on outcome. Hepatology.

[6] Tannuri A.C., Lima F., Mello E.S., Tanigawa R.Y., Tannuri U. (2016). Prognostic factors for the evolution and reversibility of chronic rejection in pediatric liver transplantation. Clinics (Sao Paulo).

[7] Charlton M., Levitsky J., Aqel B., OʼGrady J., Hemibach J., Rinella M., Fung J., Ghabril M., Thomason R., Burra P. (2018). International Liver Transplantation Society Consensus Statement on Immunosuppression in Liver Transplant Recipients. Transplantation.

[8] Colvin R.B. (2006). C4d in liver allografts: A sign of antibody-mediated rejection?. Am. J. Transpl..

[9] Kerkar N., Lakhole A. (2016). Pediatric liver transplantation: A North American perspective. Expert Rev. Gastroenterol. Hepatol..

[10] Paterno F., Shiller M., Tillery G., O’Leary J.G., Susskind B., Trotter J., Klintmalm G.B. (2012). Bortezomib for acute antibody-mediated rejection in liver transplantation. Am. J. Transpl..

[11] Wozniak L.J., Naini B.V., Hickey M.J., Bhattacharyya S., Reed E.F., Busuttil R.W., Farmer D.G., Vargas J.H., Venick R.S., McDiarmid S.V. (2017). Acute antibody-mediated rejection in ABO-compatible pediatric liver transplant recipients: Case series and review of the literature. Pediatr. Transplant..

[12] Lee B.T., Fiel M.I., Schiano T.D. (2021). Antibody-mediated rejection of the liver allograft: An update and a clinico-pathological perspective. J. Hepatol..

[13] Shindoh J., Akamatsu N., Tanaka T., Kaneko J., Tamura S., Sakamoto Y., Hasegawa K., Sugawara Y., Makuuchi M., Kokudo N. (2016). Risk factors for acute liver allograft rejection and their influences on treatment outcomes of rescue therapy in living donor liver transplantation. Clin. Transpl..

[14] Satapathy S.K., Jones O.D., Vanatta J.M., Kamal F., Kedia S.K., Jiang Y., Nair S.P., Eason J.D. (2017). Outcomes of Liver Transplant Recipients With Autoimmune Liver Disease Using Long-Term Dual Immunosuppression Regimen Without Corticosteroid. Transplant. Direct.

[15] Demetris A.J., Bellamy C., Hubscher S.G., O’Leary J., Randhawa P.S., Feng S., Neil D., Colvin R.B., McCaughan G., Fung J.J. (2016). 2016 Comprehensive Update of the Banff Working Group on Liver Allograft Pathology: Introduction of Antibody-Mediated Rejection. Am. J. Transplant..

[16] Lunz J., Ruppert K.M., Cajaiba M.M., Isse K., Bentlejewski C.A., Minervini M., Nalesnik M.A., Randhawa P., Rubin E., Sasatomi E. (2012). Re-examination of the lymphocytotoxic crossmatch in liver transplantation: Can C4d stains help in monitoring?. Am. J. Transpl..

[17] Tambur A.R., Herrera N.D., Haarberg K.M., Cusick M.F., Gordon R.A., Leventhal J.R., Friedewald J.J., Glotz D. (2015). Assessing Antibody Strength: Comparison of MFI, C1q, and Titer Information. Am. J. Transpl..

[18] Neil D.A.H., Bellamy C.O., Smith M., Haga H., Zen Y., Sebagh M., Ruppert K., Lunz J., Hübscher S.G., Demetris A.J. (2018). Global quality assessment of liver allograft C4d staining during acute antibody-mediated rejection in formalin-fixed, paraffin-embedded tissue. Hum. Pathol..

[19] Buis C.I., Geuken E., Visser D.S., Kuipers F., Haagsma E.B., Verkade H.J., Porte R.J. (2009). Altered bile composition after liver transplantation is associated with the development of nonanastomotic biliary strictures. J. Hepatol..

[20] Corbani A., Burroughs A.K. (2008). Intrahepatic cholestasis after liver transplantation. Clin. Liver Dis..

[21] Ge X., Uzunel M., Ericzon B.G., Sumitran-Holgersson S. (2005). Biliary epithelial cell antibodies induce expression of toll-like receptors 2 and 3: A mechanism for post-liver transplantation cholangitis?. Liver Transpl..

[22] O’Leary J.G., Michelle Shiller S., Bellamy C., Nalesnik M.A., Kaneku H., Jennings L.W., Isse K., Terasaki P.I., Klintmalm G.B., Demetris A.J. (2014). Acute liver allograft antibody-mediated rejection: An inter-institutional study of significant histopathological features. Liver Transpl..

[23] Guo H., Chen Z.H., Chen Z.S., Zeng F.J., Ming C.S., Zhang W.J., Liu B., Gong N.Q., Jiang J.P., Wei L. (2011). [Histopathological study of 268 hepatic allograft biopsies]. Zhonghua Yi Xue Za Zhi.

[24] Ali S., Ormsby A., Shah V., Segovia M.C., Kantz K.L., Skorupski S., Eisenbrey A.B., Mahan M., Huang M.A. (2012). Significance of complement split product C4d in ABO-compatible liver allograft: Diagnosing utility in acute antibody mediated rejection. Transpl. Immunol..

[25] Cohen D., Colvin R.B., Daha M.R., Drachenberg C.B., Haas M., Nickeleit V., Salmon J.E., Sis B., Zhao M.H., Bruijn J.A. (2012). Pros and cons for C4d as a biomarker. Kidney Int..

[26] Wozniak L.J., Hickey M.J., Venick R.S., Vargas J.H., Farmer D.G., Busuttil R.W., McDiarmid S.V., Reed E.F. (2015). Donor-specific HLA Antibodies Are Associated With Late Allograft Dysfunction After Pediatric Liver Transplantation. Transplantation.

[27] Smith J.D., Banner N.R., Hamour I.M., Ozawa M., Goh A., Robinson D., Terasaki P.I., Rose M.L. (2011). De novo donor HLA-specific antibodies after heart transplantation are an independent predictor of poor patient survival. Am. J. Transpl..

[28] Willicombe M., Brookes P., Sergeant R., Santos-Nunez E., Steggar C., Galliford J., McLean A., Cook T.H., Cairns T., Roufosse C. (2012). De novo DQ donor-specific antibodies are associated with a significant risk of antibody-mediated rejection and transplant glomerulopathy. Transplantation.

[29] Harrington C.R., Yang G.Y., Levitsky J. (2021). Advances in Rejection Management: Prevention and Treatment. Clin. Liver Dis..

[30] Tajima T., Hata K., Okajima H., Nishikori M., Yasuchika K., Kusakabe J., Yoshizawa A., Fukumitsu K., Anazawa T., Tanaka H. (2019). Bortezomib Against Refractory Antibody-Mediated Rejection After ABO-Incompatible Living-Donor Liver Transplantation: Dramatic Effect in Acute-Phase?. Transpl. Direct.

[31] Del Bello A., Congy-Jolivet N., Muscari F., Lavayssière L., Esposito L., Cardeau-Desangles I., Guitard J., Dörr G., Suc B., Duffas J.P. (2014). Prevalence, incidence and risk factors for donor-specific anti-HLA antibodies in maintenance liver transplant patients. Am. J. Transpl..

[32] Krishnamoorthy T.L., Miezynska-Kurtycz J., Hodson J., Gunson B.K., Neuberger J., Milkiewicz P., Oo Y.H. (2016). Longterm corticosteroid use after liver transplantation for autoimmune hepatitis is safe and associated with a lower incidence of recurrent disease. Liver Transpl..

[33] Ho M.H., Wu S.Y., Ou K.W., Su T.F., Hsieh C.B. (2018). Retransplant as Rescue Treatment for ABO-Compatible Living-Donor Liver Transplant Related Antibody-Mediated Rejection: A Case Report. Exp. Clin. Transpl..

